# Wound Bed Preparation for Chronic Diabetic Foot Ulcers

**DOI:** 10.1155/2013/608313

**Published:** 2013-02-13

**Authors:** Arman Zaharil Mat Saad, Teng Lye Khoo, Ahmad Sukari Halim

**Affiliations:** Department of Reconstructive Sciences, School of Medical Sciences, Universiti Sains Malaysia, 16150 Kubang Kerian, Kelantan, Malaysia

## Abstract

The escalating incidence of diabetic mellitus has given rise to the increasing problems of chronic diabetic ulcers that confront the practice of medicine. Peripheral vascular disease, neuropathy, and infection contribute to the multifactorial pathogenesis of diabetic ulcers. Approaches to the management of diabetic ulcers should start with an assessment and optimization of the patient's general conditions, followed by considerations of the local and regional factors. This paper aims to address the management strategies for wound bed preparation in chronic diabetic foot ulcers and also emphasizes the importance of preventive measures and future directions. The “TIME” framework in wound bed preparation encompasses tissue management, inflammation and infection control, moisture balance, and epithelial (edge) advancement. Tissue management aims to remove the necrotic tissue burden via various methods of debridement. Infection and inflammation control restores bacterial balance with the reduction of bacterial biofilms. Achieving a moist wound healing environment without excessive wound moisture or dryness will result in moisture balance. Epithelial advancement is promoted via removing the physical and biochemical barriers for migration of epithelium from wound edges. These systematic and holistic approaches will potentiate the healing abilities of the chronic diabetic ulcers, including those that are recalcitrant.

## 1. Introduction 

The principles involved in wound bed treatment have influenced the management of diabetic wounds, particularly diabetic foot ulcers. Since its introduction by Falanga and Sibbald in 2000, this concept has evolved from focusing on local wound problems and management to the development of an algorithmic approach commonly summarized as the “TIME” acronym [1, 2]. In 2006, Sibbald et al. expanded the concept to look at the patient as a whole in finding the cause of the wound and optimizing general factors that impair wound healing, diabetes mellitus being one of them [3]. 

The portion of the world population with diabetes mellitus in 2003 was approximately 194 million people, and this number increased steeply to reach approximately 366 million people in 2011. The International Diabetes Federation has estimated that the world diabetes incidence will be approximately 522 million people by year 2030. This incidence has indirectly increased the prevalence of diabetic foot ulcers, which occur in 1 to 4% of patients with diabetes mellitus [4, 5], and will further contribute to minor and major amputations of the lower limbs, for which foot ulcer patients have a 25 times higher risk than the rest of the population [6].

The development of diabetic foot ulcerations is multifactorial in origin and generally due to the known consequences of diabetic mellitus, including peripheral vascular disease, peripheral neuropathy, and infection. The triad of vasculopathy, neuropathy, and immunopathy affects approximately 15% of the diabetic population at some point in their life [7, 8]. Over 50% of diabetic foot ulcers are due to peripheral neuropathy, minor trauma, and foot deformities [9]. Peripheral vascular disease due to underlying atherosclerosis affects the large- and medium-sized vessels, leading to poor arterial inflow with limb ischemia. Peripheral neuropathy is due to vascular disease occluding the vasa nervorum, endothelial dysfunction, and chronic hyperosmolarity causing nerve trunk edema as a result of increased sorbitol and fructose [10]. This peripheral neuropathy causes a loss of sensation in the foot with repetitive and unnoticed trauma, structural foot deformities with uneven plantar pressure, and joint rigidities, resulting in eventual tissue breakdown and ulceration in the foot [11]. The impaired immune response is due to reduced chemotactic effects to recruit inflammatory cells with the slowing of the healing ability and thus increase the risk of infection [12]. Clinically, other identified risk factors include poor glycemic control, long-standing diabetic mellitus, smoking, retinopathy, and male gender [13]. As the diabetic patients present unique challenges to the medical profession beyond good glycemic control, it also affects protein synthesis, tissue oxygenation, white cell function, and growth factor availability, which directly dictate the progress of the wound healing process.

## 2. General and Local Factor Considerations in the Management of Chronic Diabetic Ulcers

A multidisciplinary team approach is advocated, including endocrinologists, surgeons (orthopedic, vascular, and plastic), infection control, specialist diabetic care nurses, dieticians, podiatrists, rehabilitation and medical social workers. 

Optimizing a patient's general condition had been emphasized by Ligresti and Bo as a critical part of the treatment scheme for difficult wounds [14]. In diabetic patients, blood glucose control, dietary advice, cholesterol levels, blood pressure, and kidney function need to be in the best possible condition. The patients' education regarding their illness is also an important aspect of managing the diabetic patients. For diabetic foot ulcers, foot hygiene, regular inspection, moisturizing, and appropriate footwear are important to prevent diabetic foot infections and aid in healing ulcers.

Local factors, such as the presence of peripheral vascular disease and neuropathy, may need extra attention. Screening for vascular insufficiency, such as ankle brachial index is inexpensive and widely available but can yield false results due to vessel stiffness caused by severe atherosclerosis. Another, more accurate, noninvasive method is the toe brachial index. Referral to a vascular surgeon may be necessary in moderate and severe arterial disease for possible intervention and improvement of foot perfusion such as angioplasty, stenting, or bypass procedures. The neuropathy assessment is usually performed using a 10 gm Semmes-Weinstein monofilament for protective sensation, a neurothesiometer for vibratory perception, and a clinical examination for proprioception and reflexes. Once peripheral neuropathy is established, preventing progression is essential, and education for self care and foot care should be in place. As a general guide, in neuropathic patients, the ulcers usually develop on the plantar surface, and in vasculopathic patients, the ulcer is usually on the dorsal surface. Most of the time, the ulcers are preceded by minor trauma that goes unnoticed or persistent pressure caused by inappropriate footwear or callus formation (Table 1). 

## 3. The Approaches in Wound Bed Preparation

Once the general and local conditions have been assessed and optimized, the focus can then turn to the wound management itself by following the TIME acronym. Following this TIME framework allows the systematic assessment and management of the wound. The four approaches of wound bed preparation, which address the different pathophysiological abnormalities underlying chronic wounds, are as follows (Table 2):

1. tissue management,
2. inflammation and infection control,
3. moisture balance,
4. epithelial (edge) advancement.

### 3.1. Tissue Management

**Figure 1:**
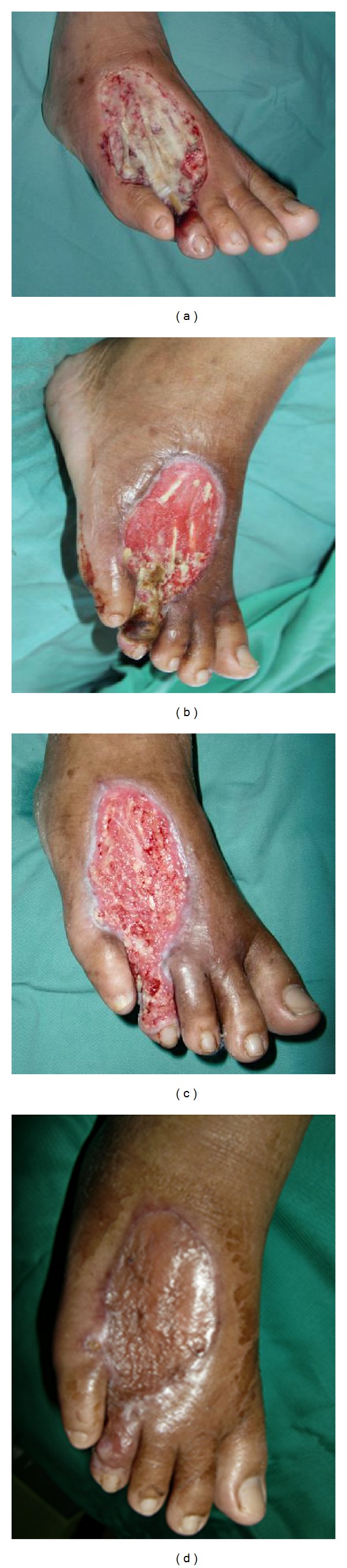
(a) A 51-year-old lady with underlying long-standing diabetes mellitus presented with large diabetic foot ulcer over her right foot dorsum, exposing extensor tendons and covered with slough tissue. (b) Regular dressings with chlorhexidine and serial bedside sharp debridement were performed to control local infection while optimizing her general and local conditions including blood sugar level. (c) Negative pressure wound therapy was applied for several cycles for wound bed pressure to achieve a vascularized wound bed covered healthy granulation tissue with advancing epithelialization. (d) The ulcer was successfully resurfaced with split skin graft and healed well without complication.

The approach to necrotic tissue in the diabetic foot needs to be given special consideration depending on the local factors, especially the tissue perfusion and the presence of active infection. In general, the presence of necrotic tissues in a wound should be removed as these tissues will obscure the underlying wound bed for proper assessment; moreover, necrotic tissue can be a source for bacterial growth and, in some instances, mask the signs of wound infection (Figure 1(a)). 

In the presence of infection, the bacterial colonies in necrotic tissue can produce unwanted metalloproteinases that negatively affect extracellular matrix components during the healing process. The bacteria may also battle for scarce local resources such as oxygen, nutrition, and building blocks that are crucial for wound healing.

Therefore, debridement of the necrotic tissue is an important component of the management of diabetic foot ulcers. There are multiple techniques that can be used for the debridement of necrotic, sloughy, fibrous, and unhealthy tissue. The options for debridement include surgical, mechanical, enzymatic, autolytic, and biological methods. 

Surgical debridement is the fastest means, allowing the surgeons to accurately assess the severity and extent of the wound. It is the method of choice, especially in life- or limb-threatening infections with necrotic eschar or gangrene. It is also indicated for wounds with extensive or adhering eschar, in which the rapid clearance of necrotic tissue is required (Figure 1(b)). The drawbacks of sharp surgical debridement are that it is nonselective, and thus normal healthy tissue may be removed at the same time [15]; bleeding; pain; and the need for anesthesia and the operating theater. However, in the presence of ischemic or neuroischemic ulcers, the management should aim toward restoring tissue perfusion prior to aggressive wound debridement or aggressive surgery to ensure wound healing and prevent the “dieback” phenomenon. Surgical debridement should be undertaken when absolutely necessary and should be performed with extreme caution to minimize damage to the healthy tissue. In some situations, when dry, noninfected gangrene, necrosis, or eschar tissue is present, they can be left in situ until autoamputation takes place, or the tissue spontaneously lifts off the wound bed. Surgical intervention may be needed if wounds turn wet or become infected.

In some centers, Versajet hydrosurgery (Smith and Nephew, Tampa, FL, USA) is used for surgical debridement of diabetic foot ulcers. This method is a technical advance in surgical debridement that uses tangential hydrodissection. This approach allows the removal of necrotic tissues layer by layer and limits the damage to the healthy tissue with its high-energy saline beam. It is also easy to operate compared to conventional surgical debridement and allow easy maneuvers in a difficult wound cavity or contours. Moreover, it is as good as the high-powered pulse lavage systems in removing bacteria [16]. It improves treatment outcomes by decreasing the number of surgeries required for the surgical wound bed preparation of acute and chronic wounds [17].

Among the nonsurgical methods of debridement, the conventional wet-to-dry dressing still has a role in the management of sloughy and minimally necrotic wounds. This technique is performed by leaving wet gauze in direct contact with wound surfaces and removing it when dried together with any adhering slough tissue. Unfortunately, this method causes excessive pain, as well as bleeding, and removes the new, healing epithelium when the dressing is changed. 

Autolytic debridement uses the inherent ability of the body to digest and remove necrotic tissue with endogenous enzymes or phagocytic cells. This approach is facilitated by a moisture-retention dressing, such as the application of a hydrogel dressing. This method is relatively easy to perform, requires limited technical skills, and involves minimal pain. It is indicated in wounds with a minimal necrotic load or that need more aggressive debridement requiring anesthesia in patients who are unable to tolerate pain [18]. However, it is time-consuming and frequently causes maceration of the surrounding skin.

Enzymatic debridement uses enzymatic agents such as collagenase and papain-urea to dissolve necrotic tissue. It is suitable for nonsurgical patients and can be effectively combined with moist wound healing. Papain is a broad-spectrum enzyme that is useful for bulk debridement, whereas collagenase is gentler on viable cells. Like autolysis-promoting agents, it can cause wound edge maceration, and additionally, the enzymatic agents are expensive.

Larval therapy has been used for the debridement of diabetic foot ulcers but still lacks properly conducted clinical trials with adequate sample sizes [19]. The larvae of the green butterfly (*Lucilia sericata*) [20] or *Lucilia cuprina* [21] can be used for biological debridement to digest the necrotic tissue, and they also secrete bactericidal enzymes. This approach is effective in wounds with methicillin-resistant *Staphylococcus aureus* (MRSA) and beta hemolytic streptococcus.

There are limited data on properly conducted clinical trials on wound debridement in diabetic foot ulcers. In the Cochrane Collaborative Review Group on the subject in 2012 [22], only six studies were eligible for analysis, of which five studies used hydrogel (one compared against larval debridement [23], three against conventional gauze dressings plus good wound care [24, 25], and one against alternative hydrogels) and one trial compared surgical debridement versus conventional nonsurgical management [26]. There were only two out of six studies to show statistically significant results for the primary outcome measured (a proportion of the complete healing of the ulcer). The two studies favored hydrogels over gauze dressing. 

### 3.2. Inflammation and Infection Control

Virtually all wounds, especially chronic diabetic wounds, contain bacteria. The level of bacteria in chronic wounds ranges from contamination, colonization, and critical colonization to infection [2]. Usually the detrimental effect on wound healing is observed in critical colonization and infection stages. In a normal person, the impact on the patient depends on three factors: the bacterial load (number of organisms); the virulence of the bacterial strain (destructive potential); and the host resistance (the capability of the patient to mount a defense). In diabetic patients, the effect of bacterial loads can be observed even at a lower count or even with the normal skin commensals due to a weak immune system and impaired leukocyte function. This situation has been associated with a higher risk of lower-limb amputation [27].

It is important to control or restore microbial balance in the wound to a state that would not interfere with wound healing. The wound infection is characterized clinically by signs such as a change in the color of the wound bed, friable and unhealthy granulation tissue, abnormal odor, increased serous exudate, and pain at the wound site. The presence of replicating microorganisms in the wound causes injury to the host due to the release of toxins, competitive metabolism, and inflammation. Systemic signs include fever, tachycardia, and even changes in mental status if sepsis occurs. The patient may have an increased white blood cell count. One must be cautious with diabetic wounds, as normal signs of infection and inflammation might not be evident, as the defense mechanisms may be defective or absent.

If bacterial colonization is suspected to affect the progression of wound healing, or wound infection is clinically suspected, local therapy using a range of antimicrobial preparation or dressings should be initiated. Deep wound swabs and/or tissue cultures should also be taken for culture and sensitivities. Infections in diabetic foot ulcers are commonly polymicrobial and contain both aerobic and anaerobic bacteria. Slow-release silver dressings have gained in popularity due to their efficacy, low resistance and broad-spectrum antimicrobial actions, and effectiveness against *Staphylococcus aureus*, including methicillin-resistant *Staphylococcus aureus* (MRSA) and pseudomonas [28]. Topical antiseptics such as aqueous chlorhexidine 0.5% and slow-release iodine have low tissue toxicity and broad-spectrum antimicrobial coverage [29]. Normal saline and chlorhexidine are suitable for most wounds as cleansing or irrigating agents due to lower toxicity to the growing new tissues. Povidone may only be considered in grossly contaminated wounds. Acetic acid may be used for pseudomonas infections. Otherwise, toxic antiseptics such as povidone, acetic acid, or hydrogen peroxide should be avoided, as they are toxic to growing dermal and epidermal cells. Wound debridement or irrigation should be performed in the presence of necrotic or sloughy tissue to reduce bacteria loads and to disrupt the biofilms that protect the bacterial from antimicrobials. Systemic antibiotics are only indicated for active wound infections, ascending cellulitis, lymphangitis, osteomyelitis, or evidence of sepsis. 

### 3.3. Moisture Balance

In general, delicate control of the wound and surrounding area moisture balance has been proven to accelerate wound healing in terms of reepithelialization, promote granulation tissue formation and prevent maceration of the surrounding skin [30]. However, in the diabetic foot, moisture control needs to be linked closely to the treatment plan, which is made based on the patient's condition. For example, in an ischemic or neuroischemic foot where there is a dry gangrene and without infection, hydration of the wound/gangrene may not be appropriate, as the gangrenous part may be converted to wet gangrene and become infected. With adequate attention, the toe, foot, or ulcer can be allowed to be dry and become mummified, thus allowing autoamputation to take place [31, 32]. 

When indicated, the wound can be kept in a balanced moisture healing environment as one would manage other chronic nonhealing wounds as part of the fundamental principles of wound bed preparation [33]. To attain moisture balance, one should create and maintain a warm, moist wound bed and avoid excessive periwound moisture that can cause surrounding skin maceration. Balanced moisture is required for the optimal effects of growth factors and cytokines within the wound to stimulate proliferating cells, such as keratinocytes, endothelial cells, and fibroblasts. Excessive moisture in the wound contains matrix metalloproteinases and serine proteases that can break down or damage essential extracellular matrix materials. The effect on surrounding skin, such as maceration, especially over the sole area, will reduce the host defensive barriers against microbial invasion provided by the thick skin in the region [34]. On the other hand, in a dry condition, cellular activities will be inhibited, an eschar will form, and further tissue necrosis may occur at the wound bed [35].

Based upon the importance of moisture balance, a vast array of dressing materials and techniques has been developed. There is no one dressing that is perfect for a chronic wound during its course of healing, as the wound healing process is dynamic. Indications for dressing materials also need to change with respect to the wound conditions. For example, available moisture-retentive dressings include occlusive, semiocclusive, absorptive, and hydrating dressings. In a highly exudative wound, an absorptive dressing such as foam will be appropriate, whereas in a dry wound eschar, an occlusive or semiocclusive dressing such as a hydrocolloid, gel-based dressing such as a hydrogel, carboxymethylcellulose, or hydroactive hydrocolloid gel will be suitable to achieve the appropriate moisture balance.

The authors use biological dressings, such as a skin allograft, which is proven beneficial in managing chronic wounds. It forms a mechanical barrier against fluid, protein, and electrolyte losses, thus preventing tissue desiccation and also microbial invasion. A skin allograft can also be used as a “take” test prior to autologous skin grafting [36, 37]. 

In heavily exudative wounds, negative pressure wound therapy (NPWT) is an important device that can be used to manage the wounds and control the moisture level. NPWT can drain away excessive wound exudates, reduce wound edema, contribute to improved tissue perfusion, and aid in reducing the wound size by promoting wound contraction and reducing the complexity of the wounds. The negative pressure exerted on the wound via the foam has microdeformation effects due to the stretching of small tissue blebs into the pores of the dressing. These stimulate changes within the cytoskeleton, resulting in cascades of biologic effects, including the stimulation of angiogenesis and formation of granulation tissue (Figure 1(c)) [38]. However, NPWT should not be used in active wound infections with excessive necrotic tissues. Instead, wound debridement should be undertaken until diminished or controlled infection before NPWT can be applied to the wound. In addition, a shorter cycle of NPWT application should be used in this wound. Eneroth and van Houtum, in their review of NPWT use in diabetic foot ulcers, found that this technique is a safe and effective treatment for complex diabetic foot wounds. It can lead to a higher proportion of healed wounds, faster healing rates, and potentially fewer reamputations than standard care [39].

### 3.4. Epithelial Advancement

One of the key indicators of a healing wound is the progression of the wound edge in terms of epidermal cell (keratinocyte) migration and wound contraction (Figure 1(c)). In a chronic diabetic foot ulcer, especially in a neuropathic ulcer, the presence of a thick callus or hyperkeratosis at the periphery of the wound will be an obstacle for keratinocyte migration and hence prevent epithelialization. At the same time, it is difficult to determine the true status of the ulcer edges, as the callus will obscure a full clinical assessment. If the patient continuously ambulates with the affected foot, pressure necrosis may develop under the callus, thus aggravating the ulcer. These adverse environments should be removed by proper debridement of all callus, slough, necrotic tissue, nonviable cellular debris, and biofilm. Pressure redistribution in the diabetic foot is important, especially in the neuropathic patient. This redistribution can be achieved by specialized or customized, prescribed footwear or with the aid of walking crutches, frame, or wheelchair to offload pressure in the foot [40]. 

Growth factor abnormalities may also cause impaired wound healing in diabetic foot ulcers. Jude et al. showed that the expression of TGF-*β*1 is not increased in diabetic foot ulcer compared to normal patients; however, TGF-*β*3 is increasingly expressed in diabetic foot ulcer subjects [41]. The expression of insulin-like growth factor (IGF)-1 is low at the edge of diabetic foot ulcer compared to a wound in a normal person [42]. The activities of basic fibroblast growth factor (bFGF)-2 are also severely reduced by the glycation process due to high sugar levels, thus impairing its ability to bind with tyrosine kinase receptor, hence the signal transduction pathway. [43]. These abnormalities are believed to cause wound healing impairment in the diabetic foot ulcer.

Liu and Velazquez have shown that local angiogenesis impairment in chronic diabetic foot ulcers is due to the inadequate presence of endothelial progenitor cells. Hyperbaric oxygen therapy enhances the mobilization of circulating endothelial progenitor cells to the wound and subsequently will promote angiogenesis, which synergistically works with the administration of exogenous stromal cell-derived factor (SDF)-1*α* [44]. There are advanced treatment modalities being used, including engineered skin constructs [45], platelet-derived growth factor [46], keratinocyte growth factor [47], granulocyte-macrophage colony-stimulating factor [48], and many more. These treatments will replace the deficient growth factors, stimulate angiogenesis, modulate inflammatory cells, promote cell proliferation, and control excessive protease activity.

## 4. Adjunct and Other Treatment Modalities for Diabetic Foot Ulcer

### 4.1. Screening

Diabetic foot screening may identify foot at risk of developing diabetic foot ulcer. There are several screening tools and classifications such as King's and Texas classifications being the most commonly used for risk assessment and ulcer classification. These classifications contained several important parameters which may account for severity or increase of the risk of ulcer development, progression, and amputation such as the presence of vasculopathy/ischemia, neuropathy, presence of infection, and depth of the ulcer [49]. Once the risk factors are identified, either neuropathic, ischaemic, or neuroischaemic feet with or without the presence of infection, appropriate actions can be taken for the prevention and also as treatment. Pecoraro et al. have shown that foot ulceration was associated with 84% of all lower-limb amputation, 61% secondary to neuropathy, and 46% due to ischaemic limb [27]. 

### 4.2. Preventive

Preventive measures for diabetic foot ulcer should be undertaken in high risk feet as it can significantly reduced the major and minor amputation rates. Myriads of strategies and interventions have been advocated to reduce the occurrences of diabetic foot ulcer which includes enhanced patient's education, intensive caretaker involvement, modified/specialized footware, pressure offloading callus debridement, bone resection, tendoachilles lengthening, neurolysis of peroneal/posterior tibial nerve neurolysis and many more [50]. There were many authors who claim the effectiveness of each modality mentioned above in cross-sectional studies, case-control studies, retrospective studies, and observational studies which are later discredited by randomized control trials [50]. However, one intervention that shows promising outcome is the plantar foot temperature-guided avoidance therapy that measures foot temperature twice a day, and if there is a difference of  >4 degrees, the patient will be instructed to contact the health care provider and instructed to reduce the activity of the affected foot until pressure gradient reduced to <4 degrees [51, 52]. The results of this technique are only presented by the same group and should be tested by a different group for the reproducibility of the results.

### 4.3. Advance Biological Therapies

The use of growth factors in nonhealing diabetic foot ulcer have been shown to increase the probability of complete healing when compared to placebo in several randomized control trials [53]. The growth factors and cytokines modulates inflammatory phases of wound healing which affect angiogenesis, cellular migration, and proliferation. The treatment needs to be administered on a daily basis to provide any desirable effect. Gene therapy can be used to modulate growth factors and cytokine production in the wound, and the effect can last up to eight weeks upon its application [54]. Mesenchymal stem cell therapy which can be obtained from the same patient have been applied to problem wounds with some success by supplying pluripotent cells to the injured tissue that develop into durable tissue and elaborate growth factors and cytokines [55]. The gene therapy and stem cell therapy can be used in combination where the stem cells act as the seed cells and carrier for the gene therapy for wound healing [56]. Bilayered living cell therapy such as Apligraf (Organogenesis, Canton, MA, USA) which contained bovine collagen and human dermal fibroblast has been shown to heal diabetic foot ulcer significantly faster than treatment with recombinant platelet derived growth factors [57]. 

### 4.4. Other Modalities

There are several other treatment modalities that are worth mentioning that has been tried and used to treat chronic wound such as laser therapy, infrared light, hydrotherapy, combination of psoralen with ultraviolet A (PUVA), radiant heat dressing, and ultrasound therapy [58]. However, there are limited data available to prove their effectiveness in clinical use at this stage [59].

## 5. Conclusion

Diabetic wound ulcers, as with any other chronic wounds, have the potential to progress through the wound healing phases without delay if the conditions of the ulcers are optimized (Figure 1(d)). Wound bed preparation is a useful approach that can help the clinician potentiate the healing ability of chronic diabetic ulcers in systematic and holistic ways. A multidisciplinary approach combining traditional and modern strategies is required when managing this challenging illness. When appropriately applied, wound bed preparation strategies could treat the most recalcitrant diabetic ulcers before any unwanted complications occur, leading to limb amputation or mortality. 

## Figures and Tables

**Table 1:** Local factors and the management.

Local factors	Effect	Aim	Management
Peripheral neuropathy	(1) Loss of pain sensation—prone to trauma	(1) Prevent progression of peripheral neuropathy	(1) Glucose control
	(2) Loss of intrinsic foot balance leading to hyperflexion of MTPJ and hyperflexion of IPJ—uneven pressure distribution	(2) Foot hygiene and prevention of trauma	(2) Appropriate footwear and podiatric advice
	(3) Charcot joint	(3) Prevention of callus formation	(3) Moisturizing
	(4) Autonomic neuropathy—lack of sweating, dry skin, and fissuring	(4) Keeping skin soft and moist	(4) Dietary supplement

Peripheral vascular disease (ABI or TBI < 0.8)	Poor foot perfusion	Restoration/optimization of tissue perfusion	Referral to vascular surgeon—angiogram, angioplasty, stenting, or bypass if possible

Inappropriate footwear—with heel, narrowed/cramped toes area	(1) Uneven pressure distribution—callosity, pressure ulceration (2) Trauma	Eliminate risk/pressure	Wear soft, fully covered shoes or sandals with back strapping, flat sole The presence of an ulcer or wound may require a special prescription shoe Treatment/removal of callus—alleviate pressure, aids advancing epithelium

**Table 2:** The summary of wound bed preparation.

	WBP Components	Problems	Aims	Actions
(1)	Tissue management	Necrotic tissue: (i) Obscuring wound assessment (ii) Nidus of bacterial infection (iii) Damaging metalloproteinases	Management of tissue necrosis: (i) Reducing necrotic tissue burden (ii) Restoring viable wound bed with functional extracellular matrix	(i) Surgical debridement (ii) Mechanical debridement (iii) Autolytic debridement (iv) Chemical debridement (v) Enzymatic debridement (vi) Biological debridement

(2)	Inflammation and infection control	Biofilm: (i) Release of bacterial toxins, competitive metabolism, and inflammation. Immunosuppression: (ii) Prone to infection	Restoration of bacterial balance	(i) Recognizing critical colonization and invasive infection (ii) Topical antimicrobial dressings (iii) Topical antiseptic solutions (iv) Wound debridement (v) Systemic antibiotic as indicated (vi) Tight control of blood sugar

(3)	Moisture balance	(i) Excessive moisture leading to maceration of wound edges (ii) Dry wound inhibits cellular activities and promotes eschar formation	Achieving balance moist wound healing environment: (i) Stimulation of actions of growth factors and cytokines, and proliferation of cells	(i) Depending on moisture status of wounds: moisture retention dressing or absorptive dressing (ii) Biological dressing such as skin allograft (iii) Negative pressure wound therapy (iv) Systemic therapy to reduce edema and control inflammation (v) Limb elevation and compression dressings if legs are edematous

(4)	Epithelial advancement	(i) Tissue necrosis as physical barrier (ii) Callus or hyperkeratosis at wound edges	Promoting migrating and intact epithelium from edges, wound contraction, and restoration of skin functions	(i) Removing necrotic tissue (ii) Removing callus and hyperkeratosis (iii) Suppression of hypergranulation (iv) Negative pressure wound therapy to promote wound contraction (v) Consider advance biological agents or skin grafting
